# Liver progenitor cells perform wound healing in a scratch assay by concerted bistable circuits

**DOI:** 10.1038/s41540-026-00724-0

**Published:** 2026-04-25

**Authors:** Marija Lazovska, Kristine Salmina, Dace Pjanova, Pawel Zayakin, Bogdan I. Gerashchenko, Jekaterina Erenpreisa

**Affiliations:** 1 Institute of Oncology and Molecular Genetics, Riga Stradins University, Riga, Latvia; 2 Latvian Biomedical Research and Study Centre, Riga, Latvia; 3 Institute of Microbiology and Virology, Riga Stradins University, Riga, Latvia; 4 R.E. Kavetsky Institute of Experimental Pathology, Oncology and Radiobiology, National Academy of Sciences of Ukraine, Kyiv, Ukraine

**Keywords:** Cell biology, Stem cells

## Abstract

Liver progenitor cells (LPC) exhibit the potential to differentiate into both hepatocytes and cholangiocytes, making them promising candidates for cell therapy in cases of severe liver pathology. However, the mechanisms of LPCs regeneration are unclear. Here, we utilised rat liver stem-like epithelial cells (WB-F344) in a wound-healing assay. The scratched near-confluent monolayer (70% area removed) underwent G1-arrest, transient bi-nucleation at 10–12 h post-injury, the epithelial-mesenchymal transition, and motion of cell fraction into the wounded areas. The transient displacement of the nuclear NANOG with upregulated p16^Ink4a^, loss of epithelial albumin and CK7 markers, along with transient YAP1/Hippo and TWIST 1 activation, was seen near the wound edge. At 24 h, G1-arrest was overcome, followed by a proliferation boost. At 40–48 h, proliferation was accomplished by reconstitution of epithelial tissue, NANOG was restored in the cell nucleus, and p16^Ink4a^ left it. Thus, wound healing was performed by concerted, molecular and cellular, bistable circuits.

## Introduction

The mammalian liver possesses a remarkable capacity for post-damage regeneration of hepatocytes. The removal of up to two-thirds of the liver (partial hepatectomy) introduced in the experiment on rats by Higgins and Anderson in 1931^1^ can be safely repaired in 48 h, by hepatocytes themselves, recovering liver volume and functionality. Hepatic diseases such as Hepatitis B and C often lead to cirrhosis and liver cancer, which represent one of the top and ascending causes of death globally^2^. For such complications, and even after surgical tumour removal, the regenerative capacity of hepatocytes becomes compromised or insufficient. Instead, the “second front” of regeneratively capable liver progenitor cells (LPCs), so-called “oval cells”, may be considered. At present, stem cell therapy is being extensively explored to tackle severe liver diseases for the replacement of the currently used liver transplantation^3^.

Although LPCs are not observed in the normal adult liver, they appear and expand in response to severe or chronic liver injury^4^. Anatomically, LPCs emerge at liver pathology, between hepatocytes and distal to the portal vein cholangiocytes of the bile duct, in the intermediate zone (known as the Canal of Hering) by de-differentiation of the biliary epithelial cells. They can differentiate into both mature hepatocytes and biliary cells^5^. In addition, hepatocytes can also de-differentiate into LPCs^6^. In rodents and humans, acute liver damage or chronic liver disease, such as late-stage cirrhosis, provokes activation of LPCs^4,7,8^. They serve as an emergency tissue compartment for the restoration of liver parenchyma^9^. Therefore, it is particularly important to clarify the mechanisms ensuring safe wound healing by LPCs for the potential cell therapy.

On the one hand, this outcome can be defined as the restitution of cell contact inhibition. Phosphorylation-induced cytoplasmic inactivation of YAP1 oncoprotein in the Hippo pathway is known to support cell contact inhibition and regulate tissue growth^10,11^. On the other hand, Hippo was shown to be involved in liver regeneration^12^. The wound healing certainly requires enhanced proliferation and cell motility events by changing cell-fate towards the epithelial-mesenchymal transition (EMT). Also, this process is known to be targeted by the Hippo pathway through the replication stress^13^ induced by nuclear import of de-phosphorylated YAP1 and induction of the EMT hallmark TWIST1, secondary to YAP1 overexpression^14^.

Accordingly, we applied here on the LPC’ rat epithelial stem-like WB-F344 cell line^15,16^ the in vitro wound healing “scratch assay” removing ~70% of the monolayer, monitoring the cell-cycle phases, along with YAP1 and TWIST1 expression and liver differentiation markers in the time-course. The Hippo pathway also restricts polyploidy and tumorigenesis^17^, while the transient polyploidy and particularly bi-nucleation through cytokinesis failure are known to be involved in liver development and regeneration^18–22^. Therefore, we also counted bi-nucleated cells at the wound edge and measured polyploidy. Finally, it is worth noting that cellular senescence is involved in cell reprogramming and regeneration, supposedly, mostly by senescence-associated inflammatory secretory phenotype (SASP)^23,24^. Moreover, recently, the pair of senescence/stemness opposite regulators, such as p16^INK4a^ and NANOG, was reported to be expressed in the neighbouring cells under stress and tumour chemotherapy^25,26^. In primitive stem cells, both of them also act as competitors, activating and blocking, respectively, the cyclin D-Cdk4,6-regulated critical G1/S transition checkpoint, with Nanog being “a propeller for stemness”^27^. Paradoxically, senescence inducer p16^INK4a^ was found to be inevitable for the proliferation capability of oval liver cells^28^.

These considerations prompted us to perform the immunofluorescent monitoring of the two opposite regulators, Nanog and p16 ^INK4a^. As a result of this study, we were able to uncover the bistable and coordinated feedback regulation in wound closure by LPCs, likely orchestrated by the Hippo pathway, with the NANOG/ p16^INK4a^ nuclear-cytoplasmic toggle operating as its concerted part.

## Results

### Cell cycle changes and the polyploidy-bi-nucleation counts at the wound edge

**Fig. 1:**
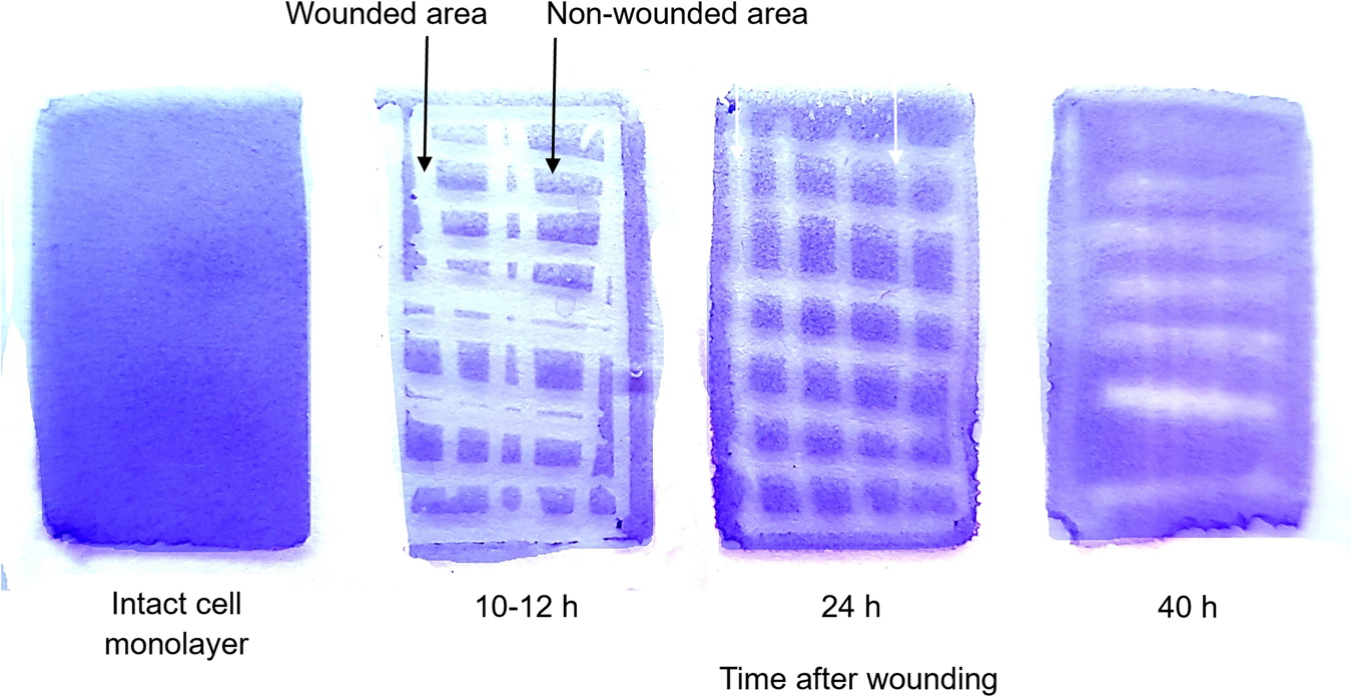
Monolayers of WB-F344 cells after reaching near 100% confluence in chamber slides were scratched, and at 40, 24, and 12 h after wounding were fixed and stained with Toluidine blue (TB) for analysis. The arrowed regions of interest from one of the TB-stained monolayers demonstrate different densities of cells in damaged (scratched) and non-damaged (non-scratched) areas, coloured in white and blue, respectively. Based on spatial localization to the wound, the edge cells were subsequently analyzed for DNA content, TB stoichiometric reaction by image cytometry, and cell-cycle phase-dependent nuclear morphology. In parallel, all four wells of the other chamber slides were stained simultaneously with the identical pair of antibodies.

Based on a series of preliminary experiments, four critical time points—hours post injury (hpi) were selected for monitoring the progression of the scratch assay (Fig. 1).

**Fig. 2:**
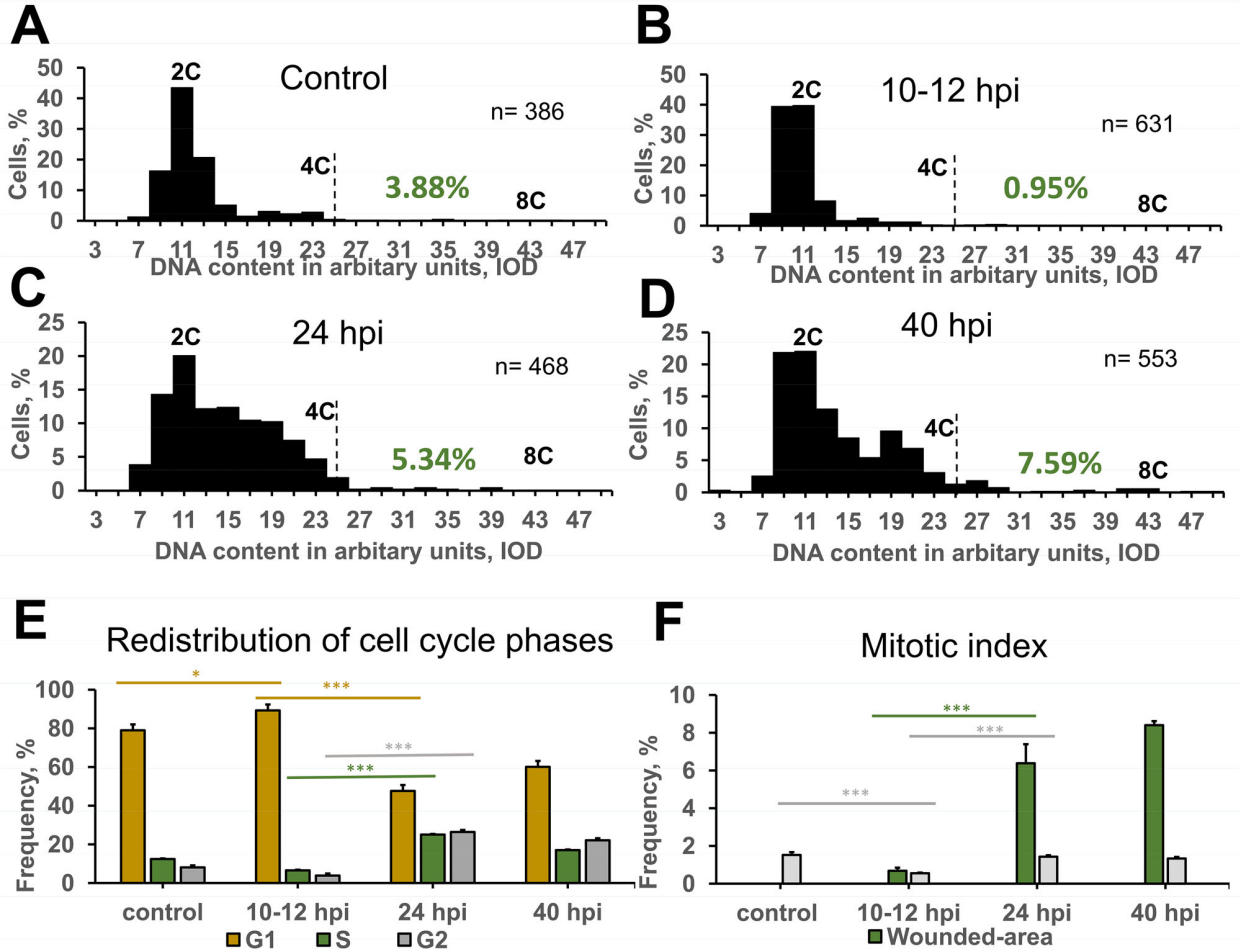
Cell cycle cytometry results during the wound healing process. Cell cycle evaluation by in situ DNA cytometry. Post-wounding changes in DNA content profiles (**A**–**D**), cell-cycle phases (**E**), and mitotic indices (**F**). **A**–**D** Representative histograms of DNA content measured by in situ image cytometry in cell nuclei of control cells (**A**) and cells at the wound edge 10–12 h (**B**) or within the gap at 24 h (**C**) and 40 h (**D**) post-wounding. DNA content is expressed as integrated optical density (IOD, arbitrary units). Dashed vertical lines indicate the 4 C DNA ploidy threshold; the percentages of nuclei exceeding this threshold are shown in green. **E** Cell-cycle phase distributions (G1, S, and G2) based on DNA content at the wound edge across different time points. **F** Mitotic indices were quantified in wounded versus non-wounded areas at each time point. Data are presented as mean ± standard error (SE) from three independent experiments. Statistical significance: ***p ≤ 0.001; **p ≤ 0.01; *p ≤ 0.05.

Cell cycle was evaluated by in situ DNA cytometry. In control, the cell cycle exhibited the distribution characteristic for normal sub-confluence with high G1, a small proportion of S-phase, and a clear G2-phase (Fig. 2A and E). Upon injury, a notable shift occurred in cell cycle dynamics registered near the wound edge at 10–12 hpi. Most cells were arrested in the G1 phase, accompanied by reduction in S- and G2 phases (Fig. 2B and E) and ~2-fold reduction of mitotic index (in both gap edge and whole population, Fig. 2F). At 24 hpi the DNA histogram shows the overcoming of the G1/S arrest accompanied by massive replication stress seen as “a ladder” starting from the early S-phase, however, still reaching the G2-phase (Fig. 2C and E) and resulting in much increased (~ 3-fold) mitotic index (Fig. 2F) indicating the start of the proliferative stage of regeneration.

At 40 hpi, the wound is closing, and the cell cycle is also closer to normality, with a clearly formed pre-mitotic G2 fraction (Fig.2D); cells still undergo high mitotic activity at the remnant wound edges (Fig. 2F). As can be judged by the changes of the cell cycle and mitotic index (Fig. 2E, F), its most significant perturbations occurred between the 10–12 hpi and 24 hpi.

**Fig. 3:**
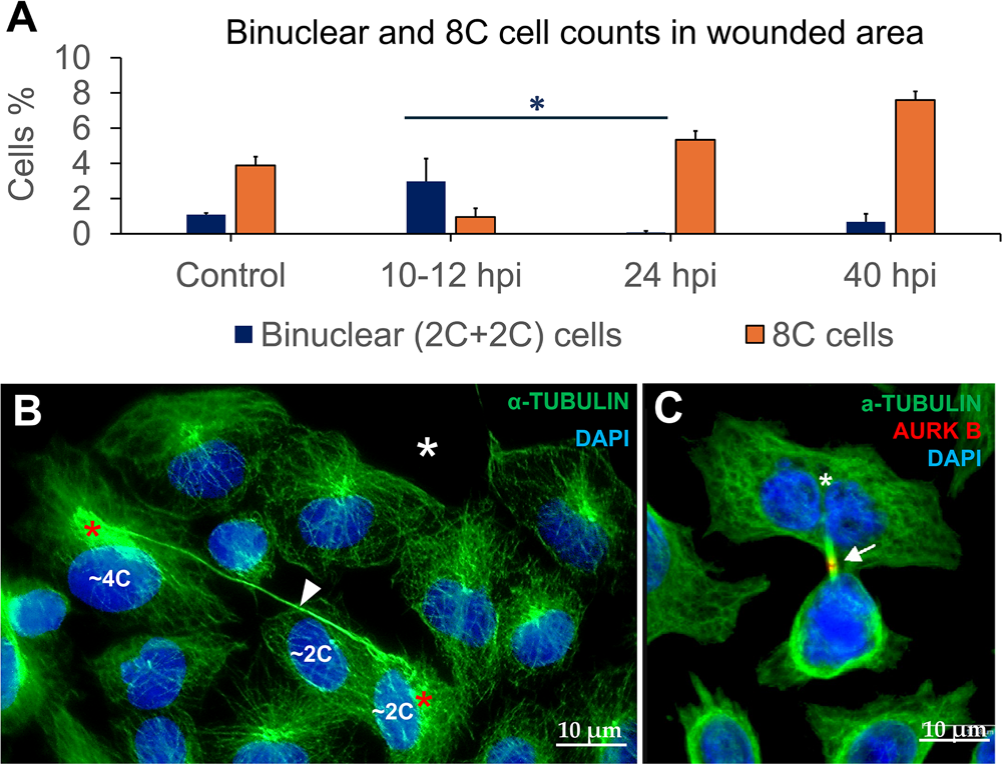
Coordinated dynamics between polyploid and binucleated WB-F344 cells during wound healing. **A** Quantification of mononuclear polyploid cells (>4C–8 C) and binucleated (largely, 2 C + 2 C) cells at the wound edge at different time points (based on DNA image analysis of individual cells). A reciprocal shift is observed: the proportion of binucleated cells increases at 12 hpi, while the 8 C population decreases, and vice versa at 24 hpi. Data are presented as mean ± standard error (SE) from three independent experiments. Statistical significance: *p ≤ 0.05. **B** Representative image at 24 hpi stained for α-tubulin (green) and DNA (DAPI, blue), showing an ~8 C binucleated cell undergoing a-cytotomic division at the wound edge (white asterisk). Two ~4 C nuclei, each with enlarged centrosomes (red asterisks), in the individual cytoplasm remained connected by a long α-tubulin bridge with a non-abscised midbody (arrowhead). A second division event shows amitosis: one ~4 C daughter underwent next binucleation into two ~2 C sub-nuclei, connected by a few microtubules. The nuclear ploidy is judged by its size. **C** The irradiated HeLa cells (10 Gy, 48 h post-irradiation) showed a similar composition: two tetraploid daughter cells are joined by the non-abscised midbody (arrow), while the upper daughter became bi-nucleated by amitotic division (white asterisk). Scale bars = 10 µm. **C** Republished from^29^.

The same critical period between 10–12hpi and 24hpi was observed in changes of polyploidy and binucleation (Fig. 3A). The proportion of mononuclear polyploid cells (>4C–8 C), ~4% in control, dropped sharply to 0.95% at the wound edge, while a small fraction of binucleated cells simultaneously tripled (from 1% to 3%). By 24 hpi, this trend reversed: the proportion of 8 C mononuclear cells increased fivefold, while binucleated cells nearly disappeared. These reciprocal changes suggest a dynamic interconversion between the two types of polyploidy.

Unexpectedly, immunofluorescence images at 24 hpi revealed the physical structure linking this circuit (Fig. 3B): cells with paired 4 C nuclei remained joined by a persistent long tubulin bridge containing the remnant of the non-abscised midbody, consistent with incomplete cytokinesis of an 8 C mother-cell. In one of the daughters, bi-nucleation was observed, with nuclei linked by scarce microtubules, thus segregated through a-mitotic splitting. It should be noted that a similar system of bi-nucleation, involving the long bridges between 4 C cell pairs, where one of the daughters was a-mitotically binucleated, was previously observed by us in irradiated HeLa cell cultures (Fig.3C)^29^.

### Biphasic response of Hippo/YAP1 pathway and tissue differentiation

**Fig. 4:**
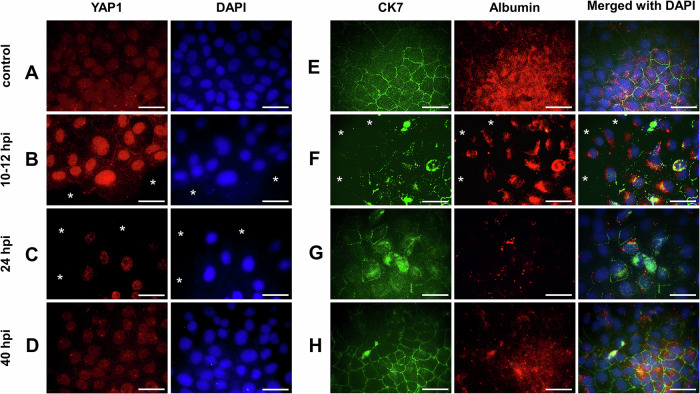
YAP1 and differentiation marker expression in WB-F344 cells at different time points after wounding. **A**–**D** YAP1 and DAPI; **E**–**H** CK7, albumin, and DAPI. **A**, **E** control; **B**, **F** 10–12 hpi; **C**, **G** 24 hpi; **D**, **H** 40 hpi. Immunofluorescence staining of YAP1 (red), DAPI (blue), CK7 (green), and albumin (red). Asterisks (*) mark the wound edge. Scale bar = 30 μm.

On Fig. 4, the typical examples of YAP1 expression and the expression of differentiation markers of WB-F344 cells at the same chosen time-points found at the wound edge are presented. In control (Fig. 4A), YAP1 is expressed in the sub-confluent culture at a low background level, while both differentiation markers albumin (hepatocytes) and cytoplasmic membrane CK7 (cholangiocytes) are well expressed and reveal a cobblestone-like epithelial pattern (Fig. 4E). However, at the 10–12 hpi (Fig. 4B), the YAP1 expression in cell nuclei of the wound edge becomes sharply increased. At the same time-point, the differentiation markers (Fig. 4F) are going down, and the tissue structure undergoes deterioration. It means that the barrier of contact inhibition at the wound edge becomes lifted. At 24 hpi (Fig. 4C), YAP1 expression in cell nuclei is still active and the epithelial tissue remains largely disordered (Fig. 4G). However, at 40 hpi (Fig. 4D), YAP1 returns to the low background level, while both differentiation markers and epithelial tissue structure are mostly restored (Fig. 4H). As well as in the cell-cycle and polyploidy change reported above, we observed a biphasic response with the critical transition between 12 hpi and 24 hpi.

### TWIST1 activation, with EMT cells in motion—at the same critical period, from 10–12 hpi to 24hpi

**Fig. 5:**
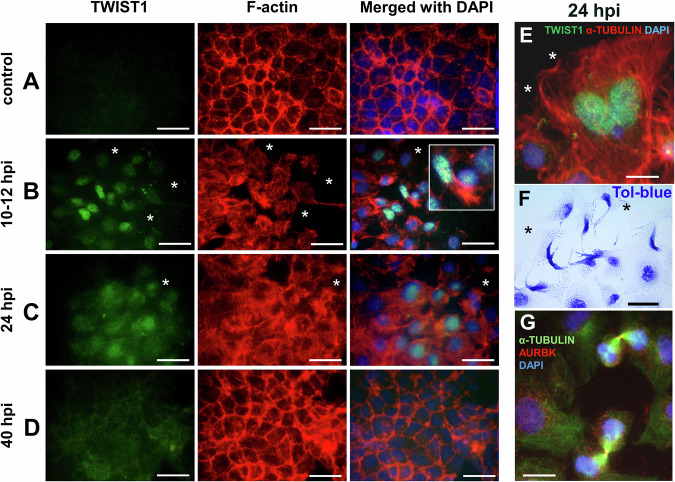
TWIST1 activation, actin remodelling, and proliferative activity at the wound edge. **A** Immunofluorescence staining of TWIST1 (green), F-actin (red), and DAPI (blue) in control and **B**, **C** wounded WB-F344 cells at 12, 24, and **D** 40 hpi; 5B, enframed - TWIST1-positive nuclei and F-actin lamellae emerge at 10–24 hpi; **E** TWIST1-positive binucleated cell at 24 hpi, co-stained with α-tubulin and DAPI; **F** A small group of motile cells at the wound edge, morphological Toluidine blue staining at 24 hpi; **G** Two cells in mitotic anaphase penetrating a wound at 24 hpi. Asterisks (*) indicate wound edges. Scale bars: 30 μm (**A**–**D**); 10 μm (**E**, **G**); 20 μm (**F**).

TWIST1 transcriptional factor, the prime epithelial-mesenchymal transition (EMT) marker, was chosen for immunocytochemical monitoring of cell motility because it is known to be directly activated by the overexpressed YAP1 protein^14^. As presented on the panel Fig. 5A-D, co-staining of TWIST1 with the F-actin (phalloidin) antibody revealed zero TWIST1 in control (A), strong nuclear activation at 10–24 hpi (B, C), and downregulation at 40 hpi (D), at the wound edge. F-actin was also activated in the cell cytoplasm at the same 12–24 hpi period. Moreover, we found some cells with positive nuclear TWIST1 extending actin lamellipodia (enframed in Fig. 5B), indicative of cell motility, emerged at 10–24 hpi. We also encountered bi-nucleated TWIST1-positive cells at 24hpi (Fig. 5E) and small groups of cells with twisted shape (Fig. 5F) at the wound edge at this stage. As well, anaphase mitotic cells were found penetrating the wound gap (Fig. 5G), in accord with proliferative boost and high mitotic index at 20–24 h post-damage. These findings fit with the period of perturbation starting wound healing registered above and underline the necessary cell motility for it, with likely involvement of the bi-nucleation and proliferation activities supporting each other.

### The nuclear-cytoplasmic toggle of the senescence and stemness regulators (p16^INK4a^ and NANOG) during the biphasic wound healing process

**Fig. 6:**
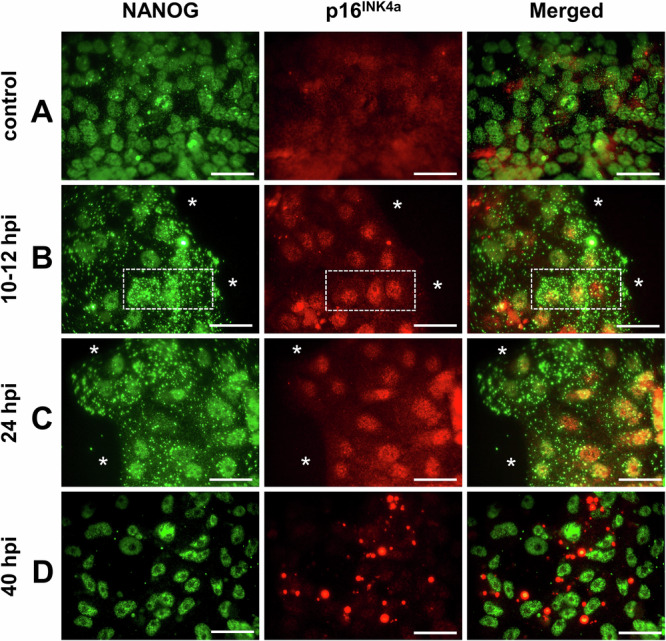
Nuclear–cytoplasmic exchange of NANOG and p16^INK4a^ during biphasic wound healing (subcellular localization patterns). Immunofluorescence staining of NANOG (green) and p16^INK4a^ (red) in WB-F344 cells: **A** at control, **B** 10–12 hpi, **C** 24 hpi and **D** 40 hpi. At 10–24 hpi, NANOG largely exits the nucleus and accumulates intracellularly in cytoplasmic granules under experimental conditions that are not observed in control cells, while p16^INK4a^ translocates into nuclei; enframed – three cells highlighting this antagonism. At 40 hpi, this switch reverses: NANOG returns to nuclei and p16^INK4a^ is excluded. Merged images show the co-localization of NANOG and p16^INK4a^. Asterisks (*) mark the wound edge. Scale bars = 30 μm.

This part of the study is illustrated by the images in Fig. 6. The stemness regulator NANOG exhibited nuclear presence in control WB-F344 cells (Fig. 6A, first column), as also reported by us earlier^30^. This baseline expression of NANOG in the absence of injury underscores the differentiation potential of these progenitor cells with stem-like features^31^.

Control cells also displayed a background level of the senescence marker p16^INK4a^ in cell nuclei and cytoplasm (Fig. 6A, second column). Upon initiation of the wound healing process, between 10–12 hpi and 24 hpi, p16^INK4a^ and NANOG displayed the nuclear-cytoplasmic exchange, as evidenced by comparative immunofluorescence images (Fig. 6B and C). p16^INK4a^ protein became overexpressed in cell nuclei and decreased its level in cytoplasm, while NANOG was abandoning nuclei (thus leaving its DNA binding sites), accumulating in the cytoplasm as multiple bright 0.2–1 µm-sized granules. These granules were consistently detected under wound-healing conditions and were strongly reduced in controls, displaying defined size, shape, and intracellular distribution, arguing against nonspecific antibody aggregation or autofluorescent lysosomal staining. Thus, senescence transiently took over stemness. Wound resolution completed at 40–48 hpi (with almost full wound closure at 40 hpi), when NANOG reinstated in cell nuclei and “cast out” p16^INK4a^ from them (Fig. 6D). Previously rich in NANOG granules, cellular cytoplasm in interphase cells is now clear from them, but the variously sized p16^INK4a^ positive cytoplasmic aggregates (2–6 µm), approximately 1-3 per cell were present instead and some were shed out of cells (sometimes colocalised with NANOG excess, not shown). Cytoplasmic p16INK4a aggregates (2–6 µm) have been reported in stress-induced senescence and autophagy-associated sequestration contexts, supporting their biological relevance rather than staining artefacts^32^. So, now recurrently, stemness took over the senescence. Interpreted as molecular correlates of the regenerative response, the observed changes in NANOG and p16^INK4a^ nuclear-cytoplasmic position reveal a recurrent shift in the balance between stemness- and senescence-associated markers, consistent with a biphasic ON/OFF–like pattern. The phase dominated by nuclear p16^INK4a^ coincided with the G1/S arrest (10–12 hpi), whereas the restoration of nuclear NANOG accompanied progression toward completion of cell proliferation.

## Discussion

The data generally correlate with in vivo liver regeneration, when performed by hepatocytes themselves, where similar three phases of the process – priming, proliferation, and termination – as well as the participation of transient binucleation have been revealed^33,34^. Interestingly, similar to the in vivo model of partial hepatectomy in rodents introduced by Higgins and Anderson^1^, the process of LPC regeneration is completed in ~48 h and includes two proliferative cell cycles. Likewise, the involvement of the Hippo pathway (with up- and down-regulation phases) has been shown as a crucial component of in vivo liver regeneration^12^; moreover, in line with our data, YAP1 was found by Camargo and colleagues to promote the expansion of undifferentiated LPCs^35^. The potential use or activation of LPCs for treating patients with severe liver pathology is on the agenda^4^, but how can their robustness and safety be ensured? Therefore, designing this study with oval cell culture in a scratch-assay, removing ~70% of cells from the nearly confluent monolayer, we sought for such mechanisms. As a result, we found the coordinated biphasic changes of the cell cycle, polyploidy, and basic markers of cell differentiation, which, after filling the wound gaps, exhibited return to homeostasis by an opposite phase.

These observations lead us to regulations by bi-stable switches (or bi-stable circuitry), a thermodynamic pillar of Systems Biology developed as Physics of Life^36^. Bistability is a crucial principle underlying cell-fate decision-making, where a biological system can oscillate between two stable “ON” and “OFF” configurations (the energy wells) acting as a molecular switch that enables all-or-none cellular decisions. It is separated by an unstable (undecided) state, which allows cells to explore the environment and by using the higher activation energy, to cross the threshold between stable functional states in response to input signals, e.g., such as those activated by cell cycle regulators^37^. Bistability allows combining flexibility with stability^38^ and represents the tightly coupled homeostasis generator and, at the same time, a driver of change^39^. It is a new paradigm in biology, where both the mechanisms of bi-stable processes used by Nature in cell-fate decisions and the methodology of their evaluation are developing.

**Fig. 7:**
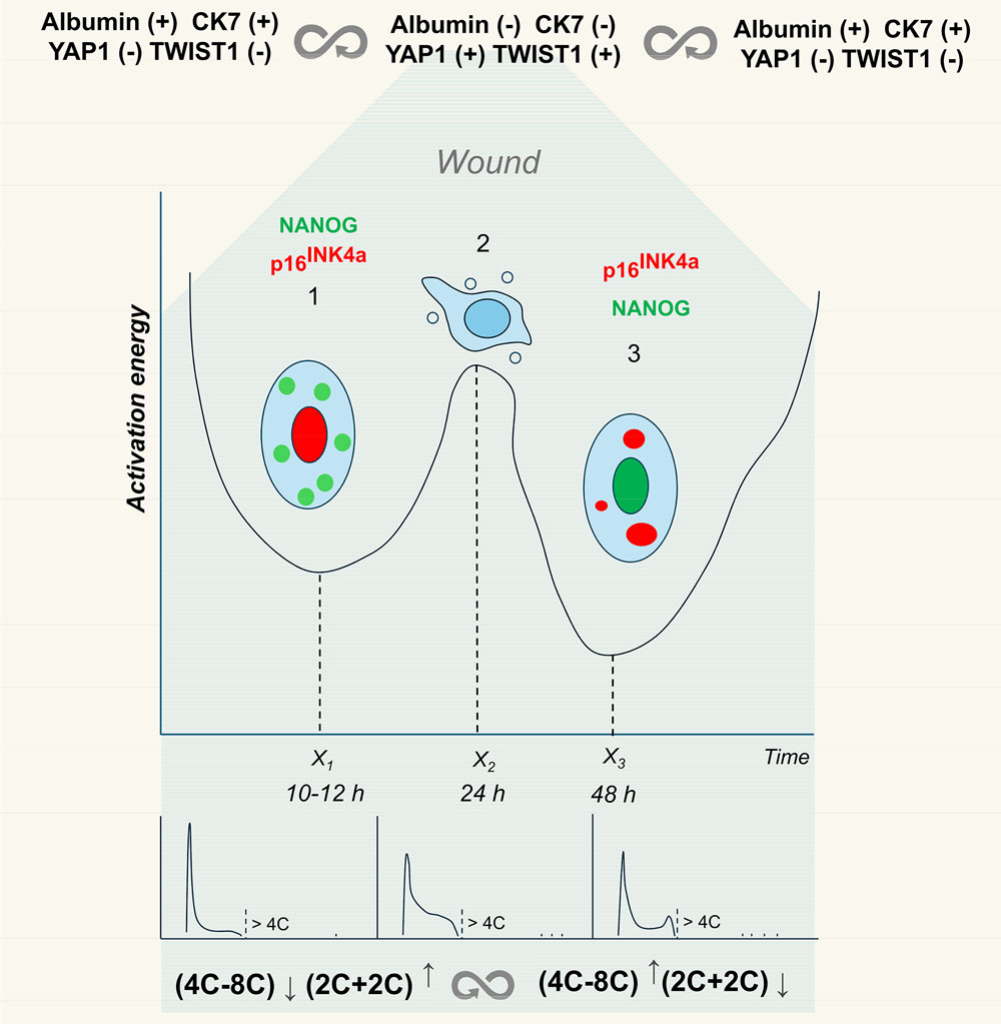
Schematic of bistable circuits during rat liver progenitor cell (WB-F344) wound healing in a scratch assay. Y axis represents Activation energy as “a latent descriptor” explaining the observed phenomenological aspects of cell-fate changes. X1,2,3 - the critical time points of these: X1–priming of wound healing: G1-arrest, loss of tissue differentiation (CK7, and albumin expression), EMT for cell motion into the wound, polyploidy store is converted into bi-nucleated cells; X2 - replication stress, energetically the highest, overcoming G1-arrest and starting the proliferation boost filling the wound by mitotically dividing cells. Senescence-Associated Secretory Phenotype is arbitrarily designed by microvesicles at the point X2; X3–energetically the lowest phase of wound healing–termination, returning population to the epithelial pattern (MET), replenishing a storage of octaploid cells, and reinstating homeostasis. The diagram also illustrates the nuclear–cytoplasmic toggle of NANOG and p16^INK4a^- the senescence-stemness pair of competitors for the G1/S replication initiation point, as part of the cell-fate change by bistable circuits, likely concerted by Hippo/YAP1 (ON/OFF) signalling.

The main components of this biphasic response in our model, embracing 48 h and the reversible cell-fate change apparent from the critical time point at 10–12 h after tissue damage are schematically presented on Fig. 7. We found in this cell line a functional tumour suppressing Hippo pathway, responsible for cell contact inhibition^10^, in the centre of the LPC response to wound healing. Hippo pathway was inactive in control, as expected for normal tissues, and became transiently activated by the wound. Notably, its main actor, transcriptional factor YAP1, is a partially intrinsically disordered protein capable of interacting and stabilizing several targets^40^, so concerting the involved processes. Moreover, YAP1 is closely linked to cellular senescence^41^.

YAP1 became expressed in cell nuclei and was kept upregulated, along with its induced TWIST1, from 10–12 hpi to 24 hpi, including as long as necessary to keep the cell motion by EMT active. Moreover, by starting the replication stress, YAP1 favoured a proliferation boost following it, as a negative feedback. In accordance with the observed, it is known that YAP1 induces the hyper-transcription which stalks the replication forks, enabling the replication stress and cell-fate change^42^. Simultaneously, at this first stage, the cells lost differentiation markers (albumin and cytokeratin 7), and the epithelial tissue structure disintegrated at the wound edge. However, between 24 and 48 hpi, both YAP1 and TWIST1 expression went down, and closer to termination, the cells reverted to the differentiation markers and restored the epithelial tissue (underwent mesenchymal-epithelial transition - MET) as depicted in Fig.7, right side.

The literature reports that interleukin-6, a pivotal senescence inducer, indispensable for reprogramming^43^, is also one of YAP1 targets^44,45^. In turn, this inflammatory cytokine is an abundant component of the senescence-induced protein secretion transferred as microvesicles. Il6 was not specifically addressed in our study, but microvesicles, as important mediators in bistability, are shown in Fig.7, time point 2. The observed changes were robustly coordinated with the changes of the cell cycle and involved the reserve of octaploid cells, which gave rise to binucleation, depicted in Fig.7, below. Bi-nucleation sharply increased but was fully exhausted by the perturbation stage, between 10–12 and 24 hpi. Bi-nuclearity was studied in the conventional liver regeneration by hepatocytes, where its origin by a-cytotomic mitosis, resolved later by normal mitosis, was reported^18–21^. Several alternative mechanisms for binucleation and increased DNA content could be considered. Cell–cell fusion appears unlikely in this in vitro LPC system, as binucleated cells consistently displayed a single continuous cytoplasm and a shared microtubule network, without multiple cortical boundaries or membrane remnants that would indicate fusion. Moreover, LPCs are of hepatic lineage, whereas fusion-mediated polyploidisation in vivo typically involves hematopoietic cells such as macrophages, a scenario not present in this culture system. Moreover, the observed configurations of two ∼4 C nuclei within one cytoplasm connected by a long, not abscised α-tubulin–positive intercellular bridge are usually characteristic of incomplete cytokinesis, a well-established mechanism of hepatocyte and LPC binucleation^18–21^. Apoptotic artefacts were also excluded, as nuclei showed smooth contours without chromatin condensation, blebbing, or micronuclei, and the cytoskeleton remained intact. Cytoplasmic continuity, microtubule organization, and nuclear morphology strongly support abrupt cytokinesis rather than fusion or apoptosis as the mechanism underlying binucleation and polyploidy in this particular model. We also noticed that the polyploidy-binucleation circuit was associated with cell motion (Twist 1-positivity) necessary to initiate filling the wound gap. At the same time, it was mostly initiated from the pre-existing 8 C cell fraction with the energetically economic means: by a-cytotomic mitosis and amitosis in the sequential formation of the binuclear cells, while the number of cells participating in this 8C-binucleation circuit was also limited. Moreover, the physical link between the members of this emergent byway is maintained by a persisting long tubulin bridge, which was found here, and may be one of the cellular mechanisms ensuring the negative feedback, which follows. This heuristic finding needs more study in the future. However, interestingly, such stabilized intercellular bridges are known from the evolution of multicellularity; they originate through arrest of the contractile cytokinetic furrow, can persist for days, and this bridge stability allows to mediate intercellular communication and transport^46^. Earlier, we observed similar mechanisms of binucleation, although embracing the majority of cells in irradiated HeLa cell cultures^29^, an example from that study is given in Fig.3C. For the role of transient binucleation, an option of the epigenetic bifurcation of cell-fates can be presumed. At the same time, this reserve polyploidy circuit, if not controlled by functional Hippo and its reciprocal cross-talk with the TP53 tumour suppressor, may give impetus or be part of malignant transformation^47^. The ploidy over 8 C and reaching 16C-32C is usually associated with conversion of mutant TP53 tumours to the phenotype of the polyploid giant cells with embryonal cancer stem cell properties^48,49^, which have the increased metastatic potential^50^. When in our previous work, the WB-F344 culture was treated by acute 1, 5, and 10 Gy ionizing radiation^30^ and examined on the 2nd day, that is at the term comparable with the term of scratch assay here, we found after 5 Gy, that the DNA histogram and polyploidy proportion, not exceeding 8 C, were very similar to that in the scratch assay. However, when 10 Gy irradiation was applied, the cells formed much more polyploid cells, reaching values of 16 C. Moreover, these LPC giants were Ki67-positive and upregulated embryonal stemness markers OCT4 and NANOG much over the levels observed after 5 Gy^30^. This data shows that the stem-like LPCs (and possibly, any tissue progenitors) can be potentially provoked towards malignancy by stress or high DNA damage, e.g., in case of over-dosage radio-chemotherapy.

Another factor, which is involved in liver regeneration by LPC is cellular senescence. It was shown that the interplay of stemness and senescence regulators in development^51^ and aging^52^ serves as a prerequisite of reprogramming (by stemness) and a barrier for cancer (by senescence)^52,53^. In the current study, we tested senescence and stemness by p16^INK4a^ and NANOG antibodies, stained in a couple. Previously, in different models, their interplay was noticed in the neighbouring cells^25,26^. Moreover, p16^INK4a^ was found paradoxically indispensable for the proliferation capability of oval liver cells^28,54^. It is noteworthy that both regulators, NANOG and p16^INK4a,^ also act in stem cells as competitors, activating and inhibiting, correspondingly, the components of the cyclin D-Cdk4,6-regulated critical G1/S checkpoint^27^. In that, they represent a typical pair of ON/OFF ‘toggle genes”—a new perspective in biological regulation widely found by transcriptome-wide scatter in multiple cell types^55^. We report here, for the first time, to our knowledge, the nuclear-cytoplasmic toggle between NANOG and p16^INK4a^ in wound healing of LPCs, which start senescence response to wound with NANOG partially leaving the cell nucleus as over competed by upregulated p16^INK4a^ and concluding this response by the opposite exchange. This event is depicted in the centre of our scheme in Fig. 7. Nuclear import and export signals were detected by many groups in OCT4, SOX2, and NANOG genes^56,57^. When the human NANOG nuclear import and export signals were first reported, the authors suggested its shuttling behaviour^56^. Stochastic NANOG fluctuations allow mouse embryonic stem cells to explore pluripotency^58^. Moreover, on the model of irradiated lymphoblastoma treated with the alpha-trans-retinoic acid suppressing *OCT4A* promoter, we previously described a similar nuclear cytoplasmic transition of NANOG particles with PML bodies^59^. So, this NANOG shuttling between the cell nucleus and cytoplasm is molecularly permissive and biologically relevant. Moreover, NANOG export allows a transient lock of the G1/S initiation checkpoint by p16^INK4a,^ which enters the cell nucleus. Contrary to this, at the return phase, p16^INK4a^ aggregates are seen expelled into the cytoplasm, while the DNA-binding NANOG returns its expression in the cell nuclei. The removal of p16^INK4a^ excess by cytoplasmic autophagy has been shown by us in the model of the etoposide-treated embryonal carcinoma^32^, and here it could occur likewise. The likely role of autophagy in the regeneration process completion should be detailed in the future. Finally, we conclude that potentially safe liver regeneration by LPCs is regulated by bistable switches, concerted and stabilized by tumour suppressors.

This study has some limitations, including the use of fixed-cell analysis rather than live-cell time-lapse imaging. However, key features such as incomplete cytokinesis, persistent intercellular bridges, and amitotic splitting represent relatively stable cellular states, making them well-suited for assessment in fixed preparations. The analysed time points were defined through preliminary kinetic experiments to capture critical stages of the cellular response with sufficient temporal resolution. Moreover, the observed time-dependent changes are internally consistent with the proposed sequence of events, supporting their interpretation as dynamically related states. The present work is primarily mechanistic and observational in nature. However, the toggle switch phenomenon happens at the level of a single cell^55^, in which each switch links two expression states (*A* or *B, i.e., low or high*), admitting a single discrete attractor state corresponding to all *A* or all *B* conditions. The choice between *A* and *B*, in the baseline symmetrical condition, is random. This state of things, at the population scale, should end up in approximately 50% *A* and 50% *B* cells that, in turn, implies the impossibility of detecting the toggle switch since the average expression values will cancel out and become part of the continuous variability. When in the presence of a transition, a ‘symmetry breaking’ happens and the two alternative A and B states no longer have the same probability and the relative frequency of A and B cells drastically shifts to an almost pure A or B condition. Here we have a phenotypic clear post-wounding discrete transition between 12 and 24 h registered by DNA content profiles, cell-cycle phases, and mitotic indices (Fig. 2). These cell-population discrete changes go hand-in-hand with a discrete transition observed in single cell protein expression (no random assortment of fluorescence but all-or-none states) made evident from the comparison between 10–12 hpi and 24 hpi frames in Fig. 4. The synchronicity between cell population features (Fig. 2) and single cell gene expression (Fig. 4) symmetry breaking events suggests a mechanistic explanation that in turn is corroborated by the coordinated dynamics between polyploid and binucleated WB-F344 cells during wound healing (Fig.3). Even in this case we observe a neat transition located between 12 and 24 h in terms of relative proportion of binucleated and polyploidy cells. Given the intrinsically all-or-none nature of bi-stable cell-fate decisions, the cellular states documented in our imaging provide sufficient evidence to support the proposed model.

The involvement of bi-stable gene regulation has previously been explored primarily through variability between individuals at the population level^60^. In contrast, this study focuses on temporal bi-stability within the same cell population, capturing how discrete gene-expression states emerge and shift during the transition toward a wound-healing phenotype. Our findings support the concept that biological regulation operates through a shift from continuous, analog control toward digital, bi-stable switching, enabling cells to make robust, all-or-none fate decisions^55^. Such regulatory logic becomes increasingly important in more complex biological systems and underlies fundamental processes such as tissue regeneration, development, and malignant transformation.

## Methods

### The cell line

The non-transformed male diploid rat liver epithelial cell line WB-F344 was kindly provided by Dr. Tomonori Hayashi (Radiation Effects Research Foundation, Hiroshima, Japan). WB-F433 epithelial LPC cell line was obtained without carcinogens and proved to be benign upon injection into syngeneic mice possessing the phenotype of “oval cells”^61,62^. It is a wild-type p53 cell line, rapidly proliferating, and potentially capable of differentiating into both hepatocytes and cholangiocytes^35^. The cells were cultured in RPMI 1640 growth medium (Sigma-Aldrich, St. Louis, MO, USA) supplemented with 7% fetal bovine serum (FBS) (Sigma-Aldrich, St. Louis, MO, USA) and 600 μg/ml L-glutamine (Gibco; Thermo Fisher Scientific, Waltham, MA, USA). Cells were incubated at 37 °C in a humidified 5% CO2 atmosphere, with complete medium replacement performed every 24 h. Cells were routinely sub-cultured after reaching 70-80% confluence every three days.

### Wound healing (scratch) assay

Before the wound healing assay, cells were grown on 4-well glass chamber slides coated with poly-D-lysine (Thermo Fisher Scientific, Waltham, MA, USA). About 70,000 cells were seeded in each chamber well with an aliquot of growth medium of 1 ml. To mimic the organ regeneration after the wounding and to test the wound-induced plasticity of WB-F344 cells, a simple wound healing (scratch) assay was applied. For this purpose, cell monolayers that were maintained in chamber slides reaching the near-confluence of ~95% after 24–30 h culturing were used. A sterile 1000 μl pipette tip was used to scratch a grid composed of longitudinal and transverse lines (~9 and 6 lines, respectively), so the distance between the closest non-intersecting lines was almost equal (~ 2 mm). Approximately two-thirds of the monolayer was removed. The experiment was adjusted so that the whole slides had four chambers, three different in the post-scratch time (10–12 h, 24 h, and 40–48 h), and the controls were fixed and stained at once. To take away cell debris, the cell monolayers were washed three times with pre-warmed culture media by gentle shaking, followed by the addition of fresh media. Cells were then incubated at the aforementioned conditions until the desired time point or full closure of the wound. The sampling time points were chosen based on live imaging of scratched monolayers (the live-cell imaging dataset generated during this study is available in the Zenodo repository at record ID 18399581), with four representative time points (Fig. S1) and the experimental design of the Toluidine Blue (TB)-stained wound healing assay shown in Fig. 1. Importantly, the 10–12 h post-injury (hpi) time point was specifically selected based on preliminary observations identifying it as the critical window of early molecular response, including nuclear activation of YAP1 and TWIST1, downregulation of epithelial markers, and morphological signs of EMT onset at the wound edge.

Based on spatial localization to the wound, the edge cells were subsequently analyzed for DNA content by TB stoichiometric reaction by image cytometry and cell-cycle phase-dependent nuclear morphology. In parallel, all four wells of the other chamber slides were stained simultaneously with the identical pair of immunofluorescent antibodies.

### Immunofluorescence staining

For immunofluorescencent staining, the chamber slides with live scratched adherent cells were drained from growth media and rinsed three times with warm phosphate-buffered saline (PBS), pH = 7.4 (Sigma-Aldrich, St. Louis, MO, USA), and afterwards a droplet of warm FBS was added to each well. Before the next manipulations, a chamber was gently pulled off from the slide, and the slides were allowed to air-dry for 30-60 s vertically. Then cells were fixed in methanol for 7 min at −20 °C, dipped 10 times in ice-cold acetone, and allowed to briefly dry. Slides were washed three times in 0.01% Tris-buffered saline/Tween 20 (TBST) for 5 min each. Subsequently, they were blocked for 40 min in 1% bovine serum albumin (BSA) in PBS with 0.05% Tween-20 (Sigma-Aldrich, St. Louis, MO, USA) at room temperature (RT). After blocking, the samples were covered with 1% BSA in PBS with 0.025% Tween-20 containing primary antibodies and incubated overnight at 4 °C in a humidified chamber. The next day, slides were washed five times in 0.01% TBST and covered with TBST containing appropriate secondary antibody in 1:300 dilution (goat anti-mouse IgG Alexa Fluor 488, goat anti-rabbit IgG Alexa Fluor 594 (Invitrogen, Carlsbad, CA, USA)) and incubated for 40 min at RT in the dark in a humidified chamber. After incubation, the samples were washed again for 1 min five times with 0.01% TBST and once for 2 min in 1xPBS. Cells were counterstained with DAPI (0.25 μg/ml) for 2 min and embedded in Prolong Gold (Invitrogen, Carlsbad, CA, USA). When staining for α-tubulin and actin, the cells were fixed with freshly prepared 4% paraformaldehyde for 15 min at RT, then washed three times with 1xPBS and permeabilised with 0.5% Triton-X100 (Sigma-Aldrich, St. Louis, MO, USA) in 1xPBS for another 5 min, washed again, and the previously described steps starting from blocking were carried out. Three independent experiments for each combination of primary antibodies were performed. For microscopic observations and sample analysis, a fluorescent light microscope (Leitz Ergolux L03-10, Leica, Wetzlar, Germany) equipped with a colour video camera (Sony DXC 390 P, Sony, Tokyo, Japan) and an RGB filter-set were used. For each distinct antibody pair microscopy configuration remained consistent during image acquisition. Primary antibodies and their sources are listed in Table 1.

**Table 1:** List of antibodies used in this study for immunofluorescence staining

Antibody Against	Description	Used concentration	Product no. and Manufacturer
α-Tubulin	Mouse monoclonal	1:200	T5168, Sigma- Aldrich, St.Louis, MO, USA
Albumin	Rabbit polyclonal	1:200	PA5-85166, Invitrogen, Carlsbad, CA, USA
NANOG	Mouse monoclonal	1:50	N3038, Sigma- Aldrich, St. Louis, MO, USA
TWIST1	Mouse monoclonal	1:100	sc-81417, Santa Cruz, Dallas, TX, USA
Cytokeratin 7 (CK7)	Mouse monoclonal	1:100	MA1-06315. Invitrogen, Carlsbad, CA, USA
p16^INK4a^	Rabbit polyclonal	1:50	ab84075, Abcam, Cambridge, UK
F-ACTIN	Phalloidin-iFlour 594 Conjugate	1:300	ab176757, Abcam, Cambridge, UK
YAP1	Rabbit polyclonal	1:500	PA-46189, Invitrogen, Carlsbad, CA, USA
Aurora B kinase	Rabbit polyclonal	1:300	ab2254, Abcam, Cambridge, UK

For consistency and clarity, all protein names are presented using the standardized human nomenclature throughout this manuscript, although the experimental data were obtained using rat cells.

### Stoichiometric in situ DNA staining and image cytometry

To evaluate DNA amount in cells, count mitotic figures, and estimate cell cycle dynamics, the samples were stained with toluidine blue (TB) using the established method^63^, by the following protocol. Chamber slides with adherent scratched or control cells were washed three times with 1xPBS as described before, with subsequent addition of warm FBS and air-drying for 30 min. Then slides were fixed in ethanol/acetone (1:1) for at least 30 min at 4 °C and air-dried again. Next, slides were hydrolyzed with 5 N HCl for 20 min at 20-21°C. For morphology, to partly preserve RNA and observe nuclei together with the cytoplasm and, in some settings, for approximate cell ploidy estimation, a shortened hydrolysis for 10 s was performed before TB staining. Slides were then washed in distilled water five times, 1 min each, and stained with 0.05% TB in 50% citrate-phosphate McIlvain buffer at pH = 4 for 10 min. They were briefly rinsed in distilled water and blotted with filter paper. Subsequently, the slides were dehydrated by incubating twice in butanol for 3 min each at 37 °C, cleared twice in xylene for 3 min each at RT, and embedded in DPX. Ready DNA samples were imaged in the green channel using a Sony DXC 390 P colour-calibrated video camera (Sony, Tokyo, Japan). From the grey-scale images, DNA content was measured in arbitrary units as the integral optical density (IOD) using Image-Pro Plus 4.1 software (Media Cybernetics, Rockville, MD, USA). IOD reflects the sum of pixel intensities within a defined nuclear area and serves as a quantitative proxy for DNA content. Cell DNA content or ploidy number was compared to IOD values for metaphases and anaphase-telophase (ratio=2) and equated to diploid (2 C) DNA values in G1 cells. A sum error of 10% was established for this TB-DNA stoichiometry method using in situ image analysis, and this interval was considered for estimating the proportional distribution of cell-cycle phases. For cell cycle measurements, 300–500 interphase cells were collected at each experimental time point. Mitotic indices were counted microscopically per 1000 cells, as well as other characteristics, such as the number of binuclear cells. For counting and analysis, wounded areas were considered those at the edge of the gap (12 hpi) and areas within a gap at later time points after the scratch was made (24 hpi and 40 hpi); cells between two scratches (area where cells were not touched during the scratching) were considered as non-wounded (for illustration, the relevant areas are highlighted on live-images in Fig. S1).

### Statistical analysis

Statistical analysis was performed with the Student’s t-test tool on Microsoft Office Excel (version 2401). Statistical significance was assessed at a *p*-value < 0.05.

## Supplementary information

Supplement_figure_1

## Data Availability

The live-cell imaging dataset generated during this study is available in the Zenodo repository at [10.5281/zenodo.18399581].

## Code availability

The live-cell imaging dataset generated during this study is available in the Zenodo repository at [10.5281/zenodo.18399581].
