# Supplementary material for: Liver progenitor cells perform wound healing in a scratch assay by concerted bistable circuits
Source: NPJ Syst Biol Appl. 2026 Apr 25;12:93. doi: 10.1038/s41540-026-00724-0 (PMC13319447; doi:10.1038/s41540-026-00724-0)
Supplement: Supplementary file 1 — Supplement_figure_1 [file 41540_2026_724_MOESM1_ESM.pdf]

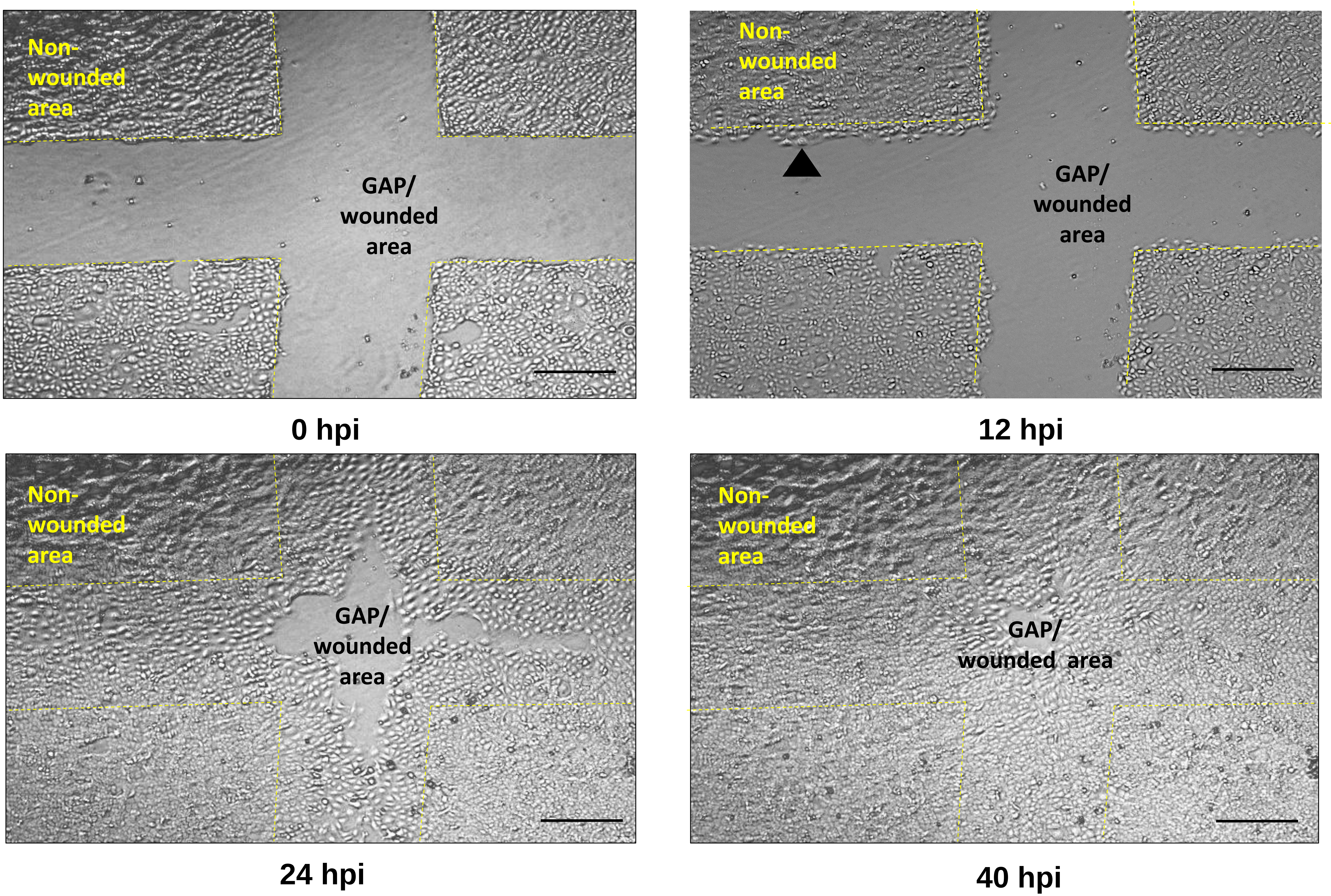

Supplementary Figure 1. Phase contrast microscopic images of WB-F344 cell scratch wounds at different time points. The yellow scattered boxes outline the initial wound site. With solid triangle are highlighted cells at the edge (beyond the yellow scattered line). Scale bars = 100 μm. hpi – hours post-injury. Three independent experiments were performed, and a representative result is shown.
